# Gastroduodenal amyloidosis: a case report and review of literature

**DOI:** 10.1093/jscr/rjab093

**Published:** 2021-04-22

**Authors:** Youssef B Almushait, Faaezuddin Syed, Safwan U Abbasi, Hussah F Alhussaini, Fahad I Alsohaibani

**Affiliations:** 1 College of Medicine, Alfaisal University, Riyadh, Saudi Arabia; 2 Department of Pathology and Laboratory Medicine, King Faisal Specialist Hospital and Research Centre, Riyadh, Saudi Arabia; 3 Department of Medicine, King Faisal Specialist Hospital and Research Centre, Riyadh, Saudi Arabia

**Keywords:** gastric, duodenum, amyloidosis

## Abstract

Amyloidosis is a disorder characterized by deposition of abnormally folded proteins in the extracellular space of various tissues and organs, possibly leading to their dysfunction. In the majority of cases, amyloidosis presents with systemic involvement including the gastrointestinal tract; however, localized gastroduodenal amyloidosis is rare. We report a case of gastroduodenal amyloidosis in a 36-year-old male with multiple comorbidities who presented with right upper quadrant abdominal pain. Reports of gastroduodenal amyloidosis and other relevant literature were also reviewed and discussed alongside this case.

## INTRODUCTION

Amyloidosis is a disease characterized by extracellular deposition of abnormal proteins in various tissues and can be classified into six types: primary (systemic), secondary (systemic), hemodialysis-related (systemic), hereditary (systemic), senile (systemic) and localized [1]. These proteins are abnormally folded and deposit as insoluble fibrils, which leads to derangement of tissue and organ function [2]. In most circumstances, amyloidosis presents with systemic involvement and the gastrointestinal tract is involved in 50–70% of the cases. However, localized gastroduodenal amyloidosis is quite rare [1, 2]. The clinical manifestations of localized gastroduodenal amyloidosis are non-specific, ranging from an asymptomatic presentation to epigastric pain, vomiting, weight loss and gastrointestinal bleeding [1, 3]. Endoscopic findings of gastroduodenal amyloidosis include; erythema, erosions, ulcerations, raised margins, nodular mucosa and polyploid lesions. The differential diagnoses for such lesions include peptic ulcers, Crohn’s disease and neoplasms such as lymphoma and gastrointestinal stromal tumors [2]. Herein, we report a case of localized gastroduodenal amyloidosis encountered at our hospital.

## CASE REPORT

A 36-year-old male presented with a history of right upper quadrant abdominal pain for the past 10 days. The patient denied other symptoms such as jaundice, changes in urine or stool color and gastrointestinal bleeding. His past medical history was significant for end-stage renal disease on hemodialysis, paraplegia due to old motor vehicle accident, neurogenic bladder on suprapubic catheter and bilateral below knee amputation due to osteomyelitis. Physical examination was significant for splenomegaly and ascites shifting dullness. Laboratory investigations revealed normocytic normochromic anemia, thrombocytopenia, elevated serum creatinine and alkaline phosphatase. Computed tomography scan of the abdomen was conclusive for splenomegaly, ascites, and diffuse edematous and thickened small and large bowel mucosa.

**Figure 1:**
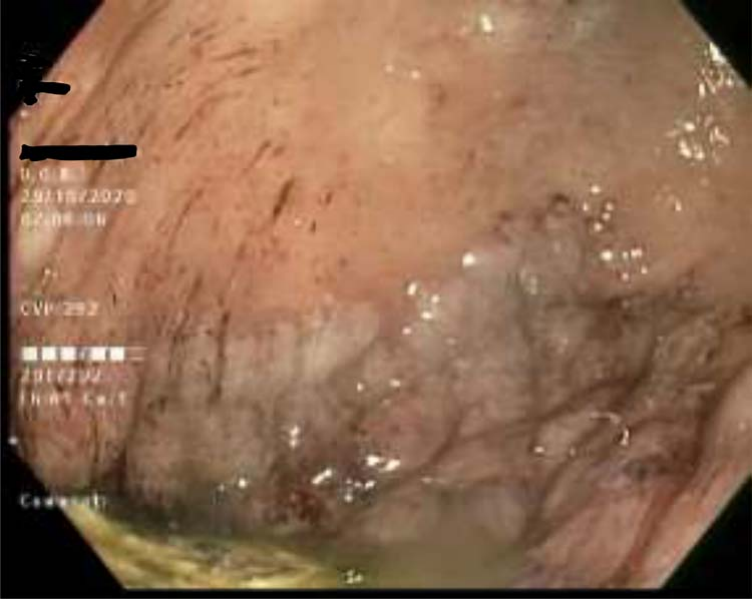
Gastric mucosa seen by endoscopy as nodular, prominent and congested.

**Figure 2:**
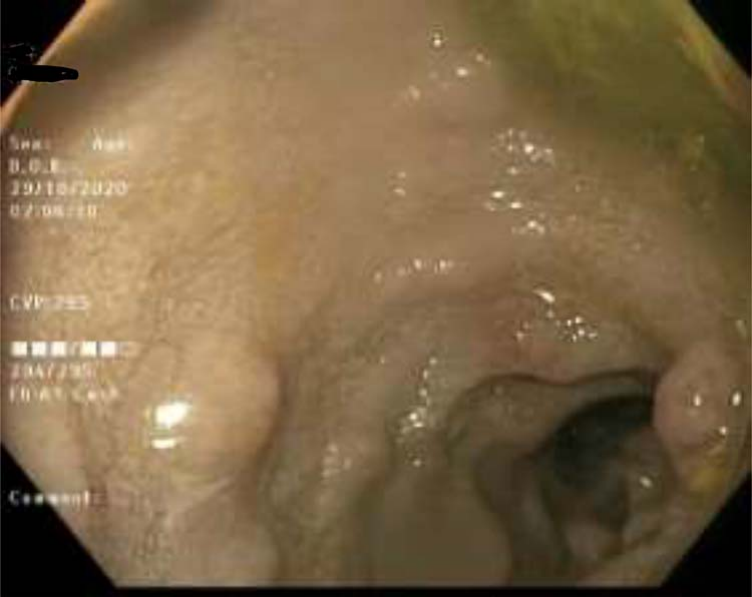
Duodenal mucosa seen by endoscopy shows some erythema and nodularity.

**Figure 3:**
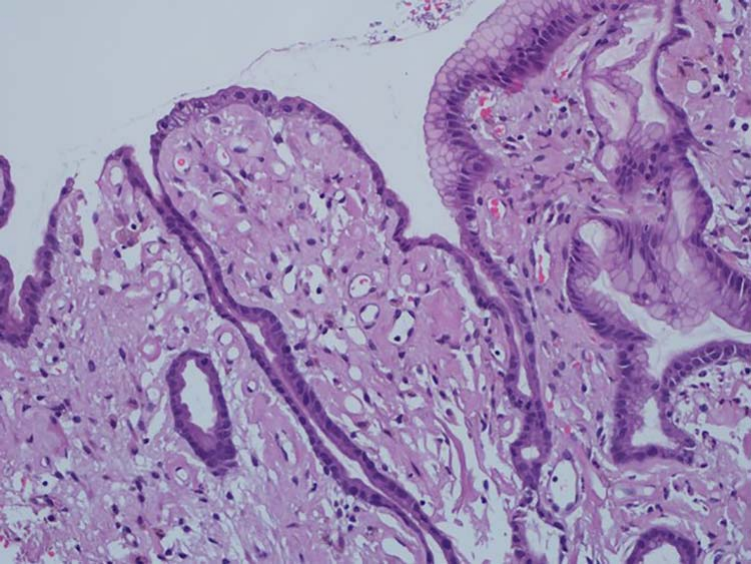
High power of gastric biopsy showing prominent stromal hyalinosis, (H&E x20).

**Figure 4:**
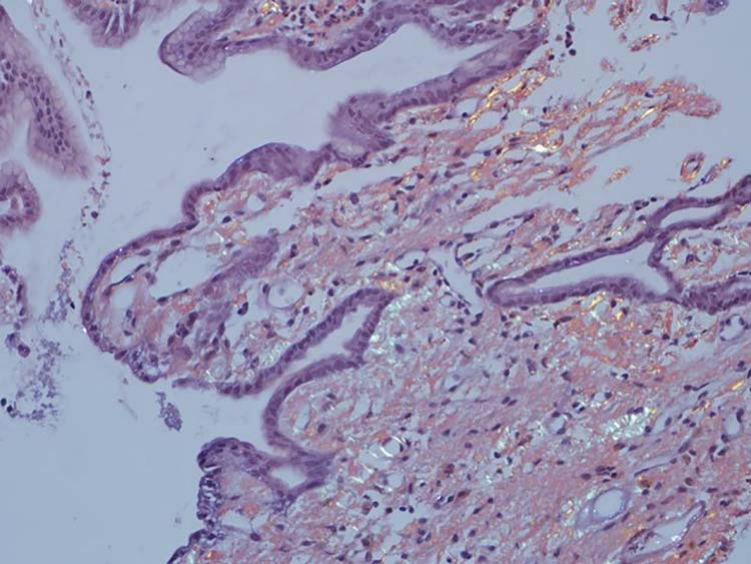
Amyloid stain (Congo red) on gastric biopsy as seen under polarized light showing apple green birefringence.

**Figure 5:**
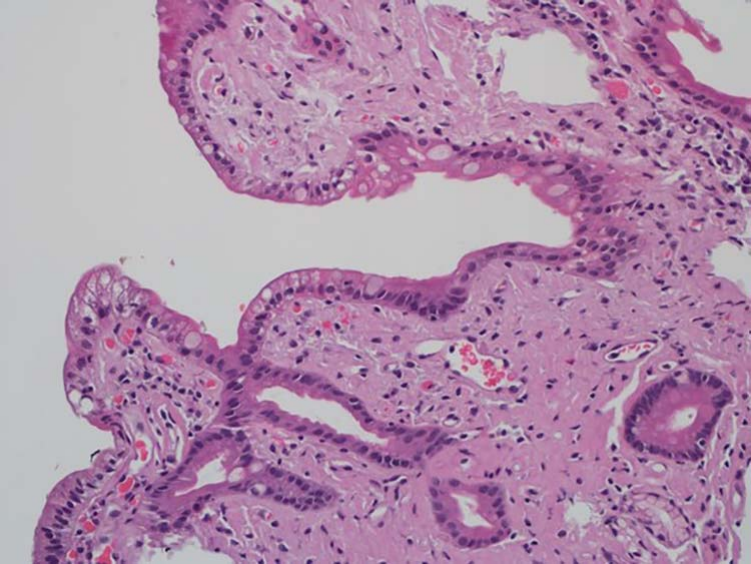
High power of duodenal biopsy showing stromal hyalinosis (H&E x 20).

**Figure 6:**
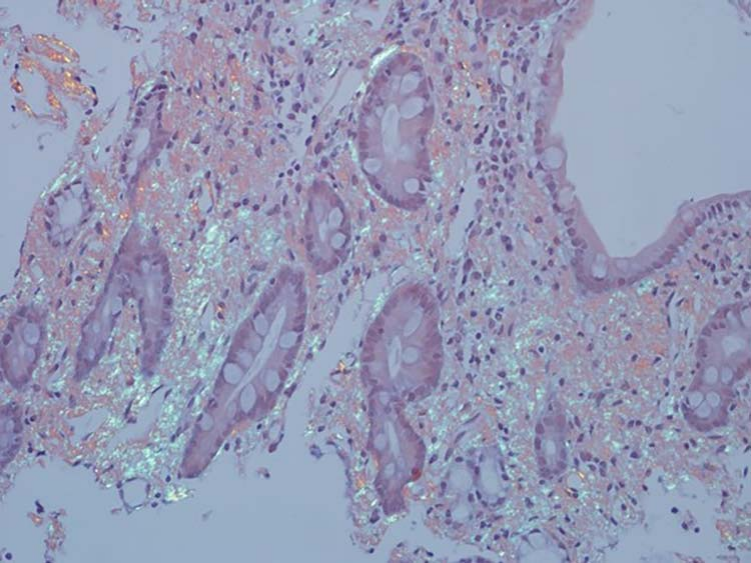
Amyloid stain (Congo red) on duodenal biopsy showing the classic apple green birefringence under polarized light.

As work up for his abdominal pain and anemia he underwent gastroscopy on 29 October 2020 that revealed congested and nodular gastric mucosa (mass-like) and at lesser extend in the first and second part of duodenum (Figs 1 and 2). Biopsies were taken from the gastric lesion and duodenum. Histopathological findings from gastric mass and duodenum showed marked stromal hyalinosis, which appeared as a cellular pink material on H&E stain (Figs 3 and 5). Congo red stain (amyloid stain) showed apple green birefringence under polarized light on both gastric and duodenal biopsies (Figs 4 and 6).

## DISCUSSION

Amyloidosis is defined as accumulation of abnormal proteins in multiple organs and tissues [4]. Amyloidosis manifests as a systemic disease and can be classified according to six forms, primary amyloid light chain (AL), secondary amyloid A (AA), hemodialysis-related B2 microglobulin (B2M), hereditary, senile and localized [4]. Theses abnormal proteins are folded and deposited as insoluble fibrils and can lead to organ failure [2]. The etiology of amyloidosis depends on the precursor protein. AL is mainly associated with occult immunocyte dyscrasia (‘primary’), multiple myeloma, macroglobulinemia or monoclonal gammopathy. AA is a reactive and associated with chronic inflammatory diseases like rheumatoid arthritis (most common, inflammatory bowel disease and autoimmune diseases or (less commonly) chronic infections and neoplastic disorders. In addition, amyloidosis can be caused by aging and hemodialysis with primary accumulation of B2M [4]. Moreover, amyloidosis is a systemic disease and involves various organs, most commonly the gastrointestinal tract.

Symptomatic amyloidosis involving gastrointestinal (GI) tract is a rare condition. In a case series of 769 patients with biopsy-proven AL amyloidosis, GI disease was noted in 8% while symptomatic stomach involvement was present in only 1% of the cases [5]. In secondary amyloidosis, about 60% of the patients can demonstrate GI involvement [5, 6]. Systemic AL amyloidosis was observed as the most common cause of GI amyloidosis with 50 of the 76 biopsy proven cases having immunoglobulin light chains [6]. It has been reported that 98% of the patients with amyloidosis will have subclinical GI disease and 50% of the patients with GI involvement will have symptomatic disease [7].

Amyloidosis occurs clinically in 0.9% of patients with Crohn’s disease and in 0.07% of patients with ulcerative colitis, although the prevalence is higher in autopsy data. It is associated with suppurative complications, found primarily in Crohn’s disease, usually taking about 15 years to develop [8].

The clinical presentation of symptomatic GI amyloidosis is dominated by malabsorption, abdominal pain, weight loss, constipation, vomiting and GI bleeding. In patients with GI involvement the presenting symptoms might tell us about the amyloid type. In AA amyloidosis, malabsorption is mostly seen due to the mucosal infiltrate seen after chronic inflammation, while in AL amyloidosis, heartburn and GI bleeding are the main presenting symptoms [9].

In AL amyloidosis, serum and urine should be tested for monoclonal light chains, which are found in 89% of patients by immunoelectrophoresis with immunofixation. The latter is used so as not to miss a small M spike. A bone marrow aspirate and biopsy should be performed to quantify the number of plasma cells and establish whether they are monoclonal. Endoscopy can also be used in the work-up; however, findings are not specific. In gastric involvement endoscopy findings can mimic gastric malignancy with ulcers, and erosions [2]. Results of gastroduodenal biopsies correlate well with other organs biopsy such as renal biopsies. The positivity of GI tract biopsies increases if submucosal vessels are sampled.

Demonstration of characteristic green birefringence under cross-polarized light following Congo-red staining of biopsied tissue remains the gold standard for confirming amyloidosis. Immunohistochemistry performed on biopsy samples is important, since it impacts treatment. It identifies all cases of AA amyloid, but AL amyloid has typically been difficult to diagnose, although a highly specialized pathologist made the diagnosis in majority of cases. Unfortunately, the test was not available at our center to be performed in our patient.

The management of amyloidosis in these patients depends on the amyloid subtype and extent of involvement determine the therapeutic regimen. AL amyloidosis is treated with chemotherapy or hematopoietic stem cell transplantation to eliminate plasma cell clones. For AA amyloidosis, control of the underlying inflammatory disorders can lead to reduction in disease progression. In AA amyloidosis associated with Crohn’s disease, Anti-Tumor Necrosis Factor (Anti-TNF) agents have resulted in some clinical improvement [10]. However, supportive measures remain the mainstay of treatment for the gastrointestinal symptoms of this disease [4]. The prognosis of gastroduodenal amyloidosis is largely dependent on the amyloid type and the extent of organ involvement.

## CONFLICT OF INTEREST STATEMENT

The authors declared no potential conflicts of interest with respect to the research, authorship, and/or publication of this article.

## FUNDING

The authors received no financial support for the research, authorship and/or publication of this article.

## ETHICAL APPROVAL

Our institution does not require ethical approval for reporting individual cases or case series.
